# Depression and Social Support Among Hospitalized Patients with Traumatic Spinal Cord Injury: A Prospective Cohort Study

**DOI:** 10.3390/healthcare14060779

**Published:** 2026-03-19

**Authors:** Badriya K. Al Shamari, Tulika Agarwal, Ayman El-Menyar, Ammar Al-Hassani, Ahammed Mekkodathil, Hassan Al-Thani

**Affiliations:** 1 Department of Clinical Administration, Hamad Medical Corporation, Doha P.O. Box 3050, Qatar; badriyakh@hotmail.com; 2 Department of Surgery, Psychology Service, Trauma Surgery, Hamad Medical Corporation, Doha P.O. Box 3050, Qatar; tagarwal@hamad.qa; 3 Department of Surgery, Clinical Research, Trauma Surgery, Hamad Medical Corporation, Doha P.O. Box 3050, Qatar; mekkodathil@yahoo.co.uk; 4 Department of Clinical Medicine, Weill Cornell Medicine, Doha P.O. Box 24144, Qatar; 5 Department of Surgery, Trauma Surgery, Hamad Medical Corporation, Doha P.O. Box 3050, Qatar; ammar_alhassani@yahoo.com (A.A.-H.); althanih@hotmail.com (H.A.-T.)

**Keywords:** depression, spinal injury, trauma, PHQ-9, psychological support

## Abstract

**Background**: Traumatic spinal injuries (TSI) are often associated with substantial physical burden and potential psychological consequences. Early detection of depressive symptoms may be important for improving quality of life during recovery. Despite the high prevalence of injury, unique sociocultural factors affecting mental health, and the need to optimize long-term rehabilitation outcomes, there is a lack of longitudinal assessments of depression in TSI patients in this region of the MENA (Middle East and North Africa). This study aimed to examine the occurrence of depressive symptoms following TSI over a 3-month period. **Methods**: A prospective cohort study was conducted to assess the occurrence of depression in TSI patients admitted between 2019 and 2022 at the Hamad Trauma Center. Conscious patients aged 18–65 years diagnosed with TSI were included. Perceived social support was assessed using the RAND Social Support Survey (Medical Outcomes Study Social Support Survey), a validated instrument measuring multiple dimensions of social support. Patient Health Questionnaire-9 (PHQ-9), a widely validated self-administered screening tool for depressive symptoms, was utilized twice: at 2 weeks and at 3 months post-trauma to evaluate early-onset depressive symptoms and their persistence or resolution over time. **Results**: A total of 189 TSI were included. The cohort was predominantly young individuals. The most common mechanisms of injury included falls (42.1%) and motor vehicle crashes (31.1%). The mean Injury Severity Score was 16.5 ± 8.2 and the spine Abbreviated Injury Scale score was 2.4 ± 0.7. Injuries involved cervical (32.8%), thoracic (38.1%), and lumbo-sacral (6.9%) regions. A total of 32.6% underwent spinal surgery, and 9.0% experienced neurological deficits. Most patients reported emotional and informational support (69%), and 62% reported caregiving support. At 2 weeks post-trauma, patients demonstrated mild depressive symptoms, with a mean PHQ-9 score of 4.6 ± 5.1, which decreased to 2.5 ± 4.2 at 3 months. The proportion of patients screening positive for depressive symptoms (PHQ-9 ≥ 5) decreased from 39.1% (52/133) at 2 weeks to 19.5% (26/133) at 3 months, corresponding to a 19.6% absolute reduction over the follow-up period. A subset of patients reported increased feelings of depression or hopelessness and sleep disturbances at three months compared with two weeks post-trauma. **Conclusions**: Patients with TSIs experience psychological distress in the early post-injury period, with a subset screening positive for depressive symptoms. Although depressive symptom scores declined over 3 months, continued psychological screening and follow-up care remain important components of comprehensive TSI management during recovery and rehabilitation. Our results should be considered cautiously because of gender-biased findings, single center data and potential attrition bias.

## 1. Introduction

Traumatic injuries are an overwhelming experience with short- and long-term complications that may affect quality of life. Traumatic spinal injuries (TSI) refer to injuries involving the vertebrae or spinal cord, with spinal cord injuries (SCI) being the more severe type [1]. SCI is often associated with a high Injury Severity Score (ISS) on the Abbreviated Injury Scale (AIS) [2]. As a result of this kind of injury, nerve fibers are injured either partially or completely. The impact can sometimes be the most devastating traumatic life event experienced by an individual and can lead to temporary or permanent loss of functions, such as weakness or paralysis [3]. These injuries are often associated with an overwhelming burden on the patient’s quality of life [4,5]. In addition, TSI can have a profound psychological impact on the individual’s life and sometimes develop into psychological disorders. Evidence reveals a deterioration in mental health status among SCI patients [6,7], thus triggering depression and affecting their overall quality of life [8,9]. Adjusting to SCI and its associated disabilities is a lifelong process, often requiring high psychological resilience.

The annual global incidence of TSI is approximately 40 to 80 cases per million in a population, with traumatic causes such as motor vehicle crashes (MVC), falls, violent acts, and sports-related injuries responsible for up to 90% of these cases [10]. The incidence is notably higher in the Middle East and North Africa (MENA) region, primarily due to the high rate of MVCs [11]. The proportion of TSI due to MVCs is high in Saudi Arabia (80%) when compared to Qatar (39%) [12,13]. Despite the high prevalence of TSI, unique sociocultural factors that affect mental health, and the need to optimize long-term rehabilitation outcomes, there is a lack of longitudinal assessments of depression in TSI patients in this region of the Middle East.

A meta-analysis [14] reported that depression affects 22.2% of SCI patients, with a lower-bound estimate of 18.7% and an upper-bound estimate of 26.3%. If not addressed, depression may lead to adverse outcomes such as lower functional independence, more secondary complications, poorer community and social integration, and lower self-appraised health. Depression can potentially affect the patient’s motivation and ability to adapt to the physiological changes that occur after acute SCI and then may compromise the patient’s rehabilitation program [15]. Optimal emotional adjustment is imperative to the recovery and rehabilitation process due to the tremendous psychological energy and motivation required for an SCI patient to learn self-care, independence, and psychosocial coping skills [16,17]. Therefore, early detection of depression becomes crucial for improving the quality of life and reducing associated comorbidities. On average, individuals with SCI experience higher levels of distress and lower levels of life satisfaction [18,19].

Despite evidence confirming an increased risk of depression among individuals with TSIs, data on the prevalence of depression across different types of spinal injuries and its longitudinal course remain limited. In Qatar, spinal injuries constitute a substantial component of the trauma burden; a prior study reported that approximately 12% of trauma patients sustained spinal fractures [13]. This highlights the clinical relevance of understanding psychological outcomes within this population.

Most research on spinal injury has been conducted in high-income countries, with limited studies from the Middle Eastern region [11,20]. This regional research gap underscores the need for more studies to address the unique challenges, particularly mental health issues, faced by individuals with TSI in the region [21].

In Qatar, between 2015 and 2019, 2240 patients with TSI were treated at the Hamad Trauma Center (HTC), representing approximately 24–28% of trauma admissions (data from the HTC registry). There is wide variability in reported prevalence estimates of depression among individuals with SCIs. This variation may be attributable to differences in demographic characteristics, diagnostic criteria, assessment tools used to measure depressive symptoms, and clinical factors such as time since injury and the level and completeness of SCI. Therefore, the objectives of this study are to (1) determine the prevalence and trajectory of depressive symptoms among patients with TSI over the first 3 months following injury and (2) describe the availability of perceived social support during the recovery period. To address these objectives, a prospective cohort study was conducted among patients with TSIs treated at a Level I trauma center.

## 2. Materials and Methods

A prospective cohort study was conducted on TSI patients with or without SCI admitted between December 2019 and January 2022 at the Hamad Medical Corporation in Qatar. Conscious male and female patients aged 18–65 years were enrolled. Patients in a confused state, in critical condition, or who had a past psychiatric history of depression were excluded. In addition, patients with a history of suicidal attempts or injuries resulting from a suicide attempt were excluded. Demographic, clinical, and psychosocial data were collected through electronic medical record reviews and patient interviews conducted during hospital admission. The study was conducted in accordance with the Declaration of Helsinki, Good Clinical Practice (GCP), and within Qatar’s Ministry of Public Health laws and regulations. Ethical approval was obtained from the Medical Research Center of the Hamad Medical Corporation. All participants provided informed consent before their inclusion in the study. Personal identifiers were kept confidential to maintain participant privacy, and participants were free to withdraw from the study at any time without consequence.

All participants were enrolled after regaining consciousness (Glasgow Coma Scale [GCS] > 13) and once clinically stable and when informed consent was obtained. According to equation (N = 50 + (8 × m)) [22], sample size was calculated to be greater than (50 plus 8 times m), where m is the number of predictors available for testing the multiple correlation plus minus 20% of the subjects that might be lost to follow-up.

GCS values reported in this study reflect routine trauma care assessments conducted at the scene, upon admission to the trauma unit, and one hour post-admission, and were not used as eligibility criteria for psychological assessment. Injury severity was assessed using the Injury Severity Score (ISS), GCS, and Abbreviated Injury Scale (AIS). The scales “ISS, GCS, and AIS” have been described in previous publications [23,24]. The AIS is an anatomically based injury severity scoring system that classifies each injury by body region on a 6-point scale. The ISS assesses the combined effects of multiply injured patients and is based on the AIS. The ISS ranges from 1 to 75 and correlates with mortality, morbidity and other measures of severity. The GCS is a neurological scale that provides reliable and objective evaluation of consciousness, ranging from 3 to 15.

Social support was assessed using a standardized instrument developed by the RAND Corporation, Santa Monica, CA USA, specifically the RAND Social Support Survey Instrument, which is part of the Medical Outcomes Study (MOS) [25]. The MOS Social Support Survey is a widely recognized, psychometrically sound, and standardized tool for assessing perceived social support and is available as a public-domain instrument for non-commercial research use. The survey evaluates multiple dimensions of social support, including emotional and informational support, tangible support, positive social interaction, and affectionate support, and has been extensively used in health-related quality-of-life and clinical outcome research.

The Patient Health Questionnaire-9 (PHQ-9), a widely validated self-administered screening tool for depressive symptoms, was used to assess depression among patients with traumatic spinal injuries [26]. The PHQ-9 total score ranges from 0 to 27 and is categorized as minimal (1–4), mild (5–9), moderate (10–14), moderately severe (15–19), and severe depression (20–27). The PHQ-9 was administered at two time points: 2 weeks and 3 months post-trauma, to evaluate early depressive symptoms and their evolution over time. Patients with PHQ-9 scores ≥5 (screening positive for depressive symptoms) were referred for further clinical evaluation and psychological support. Patients who did not complete the follow-up PHQ-9 survey at 3 months were analyzed as a separate subgroup to explore potential differences or associated factors. To assess potential attrition bias, PHQ-9 item responses at two weeks were compared between patients who completed the 3-month follow-up and those lost to follow-up using the chi-square test of independence.

Statistical analysis: Descriptive statistics for categorical variables such as gender were presented as frequencies and percentages. Numerical variables, such as age and ISS, were presented as mean and standard deviation (SD) for normally distributed data or median and Interquartile Range (IQR) for skewed data. Comparisons between groups were conducted using the chi-square test for categorical variables and Student’s t-test for continuous variables, as appropriate. Changes in depressive symptoms over time were evaluated using both continuous PHQ-9 scores and the screening threshold (PHQ-9 ≥ 5). Changes in the continuous PHQ-9 score between two weeks and three months were assessed using a paired t-test, with results reported as mean differences and 95% confidence intervals. Effect size for the change in PHQ-9 score was calculated using Cohen’s d for paired samples. Paired changes in the proportion of patients with PHQ-9 ≥ 5, as well as paired responses for individual PHQ-9 items, were assessed using McNemar’s test. Statistical significance was set at *p* < 0.05. All statistical analyses were performed using IBM SPSS, Version 23.0 [27], and results were reported with 2-tailed *p*-values to assess the significant difference.

## 3. Results

The results are presented sequentially, beginning with patient demographic and injury characteristics, followed by clinical outcomes, perceived social support, and changes in depressive symptoms assessed using PHQ-9 at two weeks and three months after trauma.

### 3.1. Patient Demographic and Injury Characteristics

**Figure 1:**
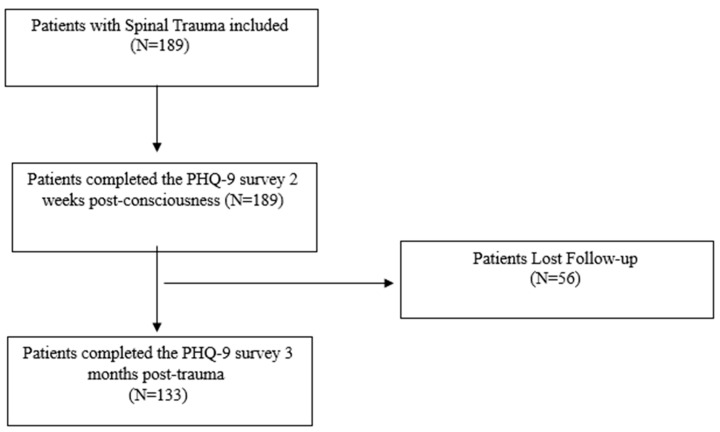
Flowchart of study process.

A total of 189 spinal trauma patients were included in this cohort study (December 2019 to January 2022); Figure 1 shows the flowchart of the study process. The demographic and clinical characteristics of the participants are summarized in Table 1. The cohort was predominantly composed of younger individuals, with the majority being between 30 and 39 years old (42.2%). Most study participants were males (93.2%). Most participants were Southeast Asian (67.4%), followed by patients from the Middle East (17.9%), Africa (13.7%), and Europe (1.0%). A small minority (4.7%) reported having no social support. Most patients had completed some level of schooling, with 58.5% having attended school, 18.9% having a university education, and 2.1% holding a diploma. A minority of patients (20.5%) were uneducated. Most participants were married (69.5%), with 30.5% of patients being single. Most participants had between 1 and 3 children (51.1%), followed by 37.4% of patients with no children. Falls were the most common mechanism of injury (42.1%), followed by motor vehicle crashes (31.1%). Most patients reported having available social support both within and outside the country (71.7%). Approximately one-third of the cohort had at least one comorbidity (31.1%). Detailed distributions are presented in Table 1.

### 3.2. Clinical Characteristics and Outcomes of Patients with Spinal Trauma

Clinical characteristics and outcomes of the cohort are summarized in Table 2, which outlines key aspects of patients’ injury severity, management, and medical needs. The average ISS for the cohort was 16.5 ± 8.2, indicating a moderate to severe injury severity across the group. The mean Injury Severity Score and GCS at the scene were 16.5 ± 8.2 and 14.3 ± 1.9, respectively. The GCS slightly decreased to 14.0 ± 2.9 upon arrival in the trauma unit. After one hour in trauma care, the GCS further decreased to 13.6 ± 3.7, suggesting some deterioration in consciousness following the injury or secondary to medications. The mean AIS score for the spine was 2.4 ± 0.7, reflecting a mix of moderate to severe spinal injuries. The breakdown of spinal injuries by region is as follows: 32.8% (n = 62) had cervical spine injuries, 38.1% (n = 72) had thoracic spine injuries, and 6.9% (n = 13) had lumbosacral spine injuries. A total of 32.6% of patients (n = 61) underwent spinal surgery, and 9.0% (n = 17) experienced neurological deficits. These deficits were identified either on initial assessment or following surgery.

Additionally, 9.0% of patients (n = 17) were referred to for psychiatric evaluation, reflecting concerns about potential mental health impacts after spinal trauma. Approximately one-third of patients (33.3%, n = 63) were referred to rehabilitation, indicating the need for ongoing physical therapy and support in recovery. Psychological care was also a critical part of patient management, with 24.3% of patients (n = 46) being referred to psychological services to address mental health concerns. The median Length of Stay (LOS) for the cohort was 10 days, with an Interquartile Range (IQR) of 5–17 days, suggesting variability in the duration of hospital care depending on the severity of the injury and recovery process.

### 3.3. Social Support Among Spinal Trauma Patients

Table 3 presents the results of the Social Support Survey, which assessed various dimensions of social support among spinal trauma patients. Most patients reported having someone they could rely on for emotional and informational support. Specifically, 69.5% (n = 132) indicated that they had someone to count on to listen when needed, while 67.9% (n = 129) had someone who provided information to help them understand their situation. Similarly, 68.4% (n = 130) had someone to give them good advice about a crisis, and 67.4% (n = 128) had someone to confide in or talk to about personal problems. A substantial proportion of patients also had practical support, with 66.8% (n = 127) reporting someone whose advice they valued and 67.4% (n = 128) having someone to share their most private worries and fears with. Furthermore, 68.9% (n = 131) had someone to turn to for suggestions on how to deal with personal problems, while 62.6% (n = 119) had someone who understood their problems. Regarding caregiving, 62.1% (n = 117) of patients had someone to help them if confined to bed, and 62.6% (n = 118) had support for transportation needs, such as going to the doctor. Additionally, 62.1% (n = 117) of patients had someone to prepare meals for them if necessary, and 58.4% (n = 110) reported having someone to help with daily chores when they were sick. When asked about emotional and social companionship, most patients reported strong support. Specifically, 77.9% (n = 147) had someone who showed them love and affection, and 80.5% (n = 152) had someone who made them feel wanted. A smaller but still significant proportion (75.8%, n = 143) had someone who hugged them. Additionally, 61.6% (n = 116) had someone to enjoy leisure activities with, such as having a good time or relaxing together (63.7%, n = 120). Furthermore, 58.4% (n = 110) had someone to do enjoyable things with, and 62.6% (n = 118) had someone to help take their mind off things.

### 3.4. Clinical Characteristics and Outcomes of Patients: Who Did Not Complete the PHQ-9 Survey 3 Months Post-Trauma

Table 4 provides a summary of the clinical characteristics and outcomes for the 56 patients who did not complete the PHQ-9 survey three months following their spinal trauma. The mean ISS for this subgroup was 15.9 ± 7.8, indicating moderate to severe trauma. The mean GCS at the scene was 14.1 ± 2.2, which decreased slightly to 13.8 ± 3.2 upon arrival at the trauma unit and further declined to 13.5 ± 3.8 after one hour in trauma care, suggesting a trend of deterioration in consciousness post-injury or medication received in TRU. Similar to the overall cohort, the majority of patients in this group had thoracic spine injuries (46.4%, n = 26) and cervical spine injuries (30.4%, n = 17). Fewer patients sustained lumbo-sacral injuries (3.6%, n = 2). The mean AIS score for the spine was 2.4 ± 0.8, reflecting moderate to severe injuries in this subgroup. Among the 56 patients, 23.2% (n = 13) underwent spinal surgery, and 10.7% (n = 6) experienced neurological deficits. A smaller proportion (12.5%, n = 7) was referred to for psychiatric evaluation, indicating the need for a mental health assessment following their injury. Additionally, 30.4% (n = 17) of patients were referred to rehabilitation, and 23.2% (n = 13) were referred to psychological services, underscoring the importance of physical and mental care in recovery. The median LOS for these patients was 8 days, with an IQR of 5–16 days, suggesting variability in the duration of hospital care required, likely dependent on the severity of the injury and the need for recovery or stabilization prior to discharge.

### 3.5. Patient Health Questionnaire (PHQ-9) Assessments Post 2 Weeks and 3 Months of Spinal Trauma

At 2 weeks post-trauma, the mean PHQ-9 score was 4.6 ± 5.1, with a median score of 3 (IQR: 1–8), indicating generally mild depressive symptoms. At 3 months, the mean PHQ-9 score decreased to 2.5 ± 4.2, with a median of 1 (IQR: 0–4). Paired analysis demonstrates a mean reduction of 2.10 points (95% CI: 1.24–2.95; *p* < 0.001). The corresponding effect size was small-to-moderate (Cohen’s d = 0.42), indicating a modest reduction in depressive symptom burden over the follow-up period. The proportion of patients screening positive for depressive symptoms (PHQ-9 ≥ 5) decreased from 39.1% (52/133) at two weeks to 19.5% (26/133) at three months, corresponding to a 19.6% absolute risk reduction. This change is statistically significant using McNemar’s test (*p* = 0.001). Detailed item-level PHQ-9 responses are presented in Table 5.

Baseline depressive symptoms at 2 weeks were compared between patients who completed the 3-month follow-up and those lost to follow-up. No statistically significant differences were observed across PHQ-9 item responses between the two groups (all *p* > 0.05), suggesting that attrition was unlikely to introduce significant bias in the longitudinal analysis (Table 6 and Table 7).

These findings provide insight into the early psychological burden experienced by patients with TSIs and the evolution of depressive symptoms during the recovery period.

## 4. Discussion

This prospective cohort study evaluates the trajectory of depressive symptoms among patients with traumatic spinal injuries during the first three months following injury. The findings indicate that depressive symptoms were relatively common in the early post-injury period, with a proportion of patients screening positive for depressive symptoms shortly after trauma. Overall, depressive symptom scores declined during follow-up, suggesting a general reduction in symptom burden over time; however, recovery trajectories were not uniform. While many patients reported improvement, a subset continued to experience persistent or emerging depressive symptoms during the follow-up period, reflecting variability in psychological adjustment after spinal trauma.

**Clinical Characteristics and Injury Severity**: The clinical profile of patients with TSIs in this cohort was comparable to that reported in previous studies involving patients sustaining high-energy trauma [28,29,30]. Most patients sustained moderate to severe injuries, as reflected by the mean ISS, underscoring the substantial trauma burden associated with spinal injuries. The predominance of thoracic and cervical spine involvement observed in this study is consistent with earlier reports and reflects the vulnerability of these regions in high-impact mechanisms [11]. In addition, nearly one-third of patients require surgical intervention, highlighting the severity of injury and the frequent need for operative stabilization in this population [31]. These findings reinforce the clinical complexity of spinal trauma and provide important context for interpreting subsequent psychological outcomes.

**Social Support and Psychological Impact**: Most patients (71.7%) reported access to social support during the recovery period. However, the present study was not designed to evaluate the relationship between social support and depressive symptom outcomes. Previous studies have suggested that social support may influence psychological well-being following traumatic injuries. In the present study, social support was described as a contextual factor during recovery, but the study was not designed to formally evaluate its association with depressive symptom outcomes. The high level of perceived support observed in this cohort is consistent with research indicating that social networks are important in the rehabilitation and psychological well-being of trauma survivors [32,33,34]. The emotional and instrumental support reported by participants, such as help with transportation and caregiving, aligns with the critical role of caregivers in facilitating post-trauma recovery [35].

The psychological burden of spinal injury is well-documented, with studies indicating that individuals with SCIs are at an increased risk for mood disorders, including depression [7]. In our study cohort, despite high levels of social support, around 12.1% of patients reported feeling sad, depressed, or down following their injury, which is considered a significant rise from the pre-injury baseline based on the self-report and inclusion criteria that none of these patients had any mental health condition before the injury happened. This is consistent with the psychological impact of spinal trauma, which is often associated with increased rates of depression, anxiety, and post-traumatic stress disorder (PTSD) [19,36]. Many participants with spinal injuries struggled with depression and other mental health challenges, underscoring the need for comprehensive psychological support throughout the rehabilitation process. However, it was found that only 24.3% of patients received referrals to the in-house Trauma Psychology service and 9% received referrals to psychiatry, which brings out a gap that needs to be addressed.

Psychological and Depressive Symptoms: The PHQ-9 assessments highlight the dynamic psychological impact of TSI during the early recovery period. Overall, depressive symptom burden was mild and showed improvement over time, as reflected by a decrease in mean PHQ-9 scores from 2 weeks to 3 months post-trauma. Consistent with this trend, the proportion of patients that screened positive for depressive symptoms (PHQ-9 ≥ 5) declined from 39.1% at 2 weeks to 19.5% at 3 months, corresponding to a 19.6% absolute reduction. Despite the overall improvement in aggregate depressive symptom scores, item-level analyses revealed that specific symptoms evolved differently over time. Notably, a subset of patients reported increased feelings of depression or hopelessness and sleep disturbances at three months compared with two weeks post-trauma. These findings suggest that while some individuals may experience early emotional numbing following injury, others may develop more pronounced psychological distress as they adapt to the longer-term consequences of spinal trauma [37]. Such delayed or fluctuating psychological responses have been described previously and may reflect increasing awareness of functional limitations and uncertainty regarding recovery.

Fatigue and low energy were reported by a proportion of patients during follow-up; however, no statistically significant change was observed over time. Persistent fatigue is a well-recognized sequela of TSI and is often exacerbated by pain, sleep disruption, and psychological demands of adjustment, collectively contributing to emotional distress and impaired recovery [38,39,40]. In contrast, no significant temporal changes were observed in appetite-related symptoms or suicidal ideation, suggesting that, while certain depressive features may fluctuate, more severe manifestations of depression were relatively uncommon in this cohort. This may reflect the benefits of early psychological support and timely referral, as prior studies have demonstrated that proactive mental health assessment following spinal trauma can mitigate the risk of severe adverse outcomes, including suicidal behavior [41].

TSI represents not only a major physical health event but also a potentially distressing life experience that can affect psychological well-being during recovery, underscoring the importance of incorporating routine psychological screening and supportive care within trauma rehabilitation pathways. Despite these important findings, several limitations should be considered when interpreting the results

### Limitations and Future Directions

Approximately 30% of participants did not complete the 3-month follow-up. To assess potential attrition bias, PHQ-9 item responses at two weeks were compared between participants who completed follow-up and those lost to follow-up. No significant differences were observed, suggesting that loss to follow-up was unlikely to have substantially biased the observed symptom trajectories, although the reduced sample size may have limited statistical power.

Additional limitations should be considered. This study was conducted at a single Level I trauma center, which may limit generalizability to other settings. The cohort was predominantly male, reflecting the regional epidemiology of traumatic injuries but potentially limiting applicability to female patients. Of note prior data revealed that less than 10% of hospitalized trauma patients in Qatar are females [42]. Moreover, 85% of the country’s population are expatriates originating from over 150 countries [43].

Depressive symptoms were assessed using the PHQ-9 screening instrument rather than a diagnostic psychiatric interview and therefore reflect screening-positive depressive symptoms rather than confirmed clinical diagnoses. Baseline mental health status prior to injury and information on psychiatric treatment during follow-up were not available. In addition, although perceived social support was measured using the MOS Social Support Survey, the study was not designed to formally evaluate its association with depressive symptom outcomes.

Future research with multi-center cohorts, longer follow-up, and comprehensive psychological assessments may further clarify the trajectory of depressive symptoms and psychosocial factors following traumatic spinal injury.

Potential cultural influences on symptoms cannot be negated and can be included in future studies which should consider more extended follow-up periods and more diverse cohorts to examine how spinal trauma impacts mental health over the years.

Attrition bias was checked by comparing the 56 patients lost to follow-up with the 133 patients who completed the 3-month assessment. Using the chi-square test, no significant differences were observed in PHQ-9 item responses at two weeks between the two groups (Table 6 and Table 7). Our trauma center hired a psychologist dedicated to trauma patients from admission until referred to the psychology clinic aiming to address PTSD and depression with appropriate follow-up and management. Routine PHQ-9 screening should be implemented at 2 weeks, 3 months, and 6 months post-TSI. Mental health services should be integrated into spinal injury rehabilitation pathways from admission through outpatient follow-up. Future multicenter studies with a longer follow-up and larger female representation are needed.

Taken together, these findings provide insight into the psychological experiences of patients recovering from TSIs.

## 5. Conclusions

In this prospective cohort of patients with TSIs, depressive symptoms were common in the early post-injury period, with a notable proportion of patients screening positive for depressive symptoms shortly after trauma. Although the overall depressive symptom burden declined over the first 3 months, recovery trajectories varied, and a subset of patients continued to report persistent or emerging symptoms during follow-up. These findings highlight the importance of early psychological screening after TSI, together with ongoing monitoring beyond the acute phase. Integrating routine mental health assessment and supportive care into spinal injury management and rehabilitation pathways may help address evolving psychological needs during recovery. Our results should be considered cautiously because of gender-biased findings, single center data and potential attrition bias.

## Figures and Tables

**Table 1:** Descriptive analysis of spinal trauma patients (N = 189).

Variable	Value
**Age**	
19–29 y	51 (26.8%)
30–39 y	79 (42.2%)
40–49 y	41 (21.6%)
50–59 y	17 (8.9%)
≥60 y	1 (0.5%)
**Sex**	
Male	176 (93.2%)
Female	13 (6.8%)
**Nationality**	
Southeast Asian	128 (67.4%)
Middle Eastern	33 (17.9%)
African	26 (13.7%)
European	2 (1.0%)
**Mechanism of injury**	
Fall	79 (42.1%)
Motor Vehicle Crash	59 (31.1%)
Hit by Objects	16 (8.4%)
Pedestrian injuries	20 (10.5%)
Others	15 (7.9%)
**Social support**	
Within the country	32 (16.8%)
Outside the country	13 (6.8%)
Outside and within the country	135 (71.7%)
No social support	9 (4.7%)
**Education**	
School	111 (58.5%)
University	35 (18.9%)
Diploma	4 (2.1%)
Uneducated	39 (20.5%)
**Marital Status**	
Married	131 (69.5%)
Single	58 (30.5%)
**Number of Children**	
0	71 (37.4%)
1–3	97 (51.1%)
4–6	20 (10.5%)
7–9	2 (1.0%)
**Comorbidities**	
Yes	59 (31.1%)
No	130 (68.4%)

**Table 2:** Clinical characteristics and outcomes of patients with spinal trauma (N = 189).

Injury Severity Score (Mean ± SD)	16.5 ± 8.2
GCS at the scene (Mean ± SD)	14.3 ± 1.9
GCS in trauma unit (Mean ± SD)	14.0 ± 2.9
GCS after 1 h in trauma (Mean ± SD)	13.6 ± 3.7
Spine AIS (Mean ± SD)	2.4 ± 0.7
Cervical spine injury	62 (32.8%)
Thoracic spine injury	72 (38.1%)
Lumbo-sacral spine injury	13 (6.9%)
Spinal surgery	61 (32.6%)
Neurological deficit	17 (9.0%)
Psychiatry referral	17 (9.0%)
Rehabilitation referral	63 (33.3%)
Psychology referral	46 (24.3%)
LOS	10 (IQR 5–17)

SD: Standard deviation; AIS: Abbreviated Injury Scale; LOS: Length of Stay; IQR: Interquartile Range.

**Table 3:** Social Support Survey among spinal trauma patients (N = 189).

**Someone to count on to listen to you when you need to talk**	
None to some of the time	57 (30.5%)
Most or all of the time	132 (69.5%)
**Someone to give you information to help you understand a situation**	
None to some of the time	60 (32.1%)
Most or all of the time	129 (67.9%)
**Someone to give you good advice about a crisis**	
None to some of the time	59 (31.6%)
Most or all of the time	130 (68.4%)
**Someone to confide in or talk to about yourself or your problem**	
None to some of the time	61 (32.6%)
Most or all of the time	128 (67.4%)
**Someone whose advice you really want**	
None to some of the time	62 (33.2%)
Most or all of the time	127 (66.8%)
**Someone to share your most private worries and fears with**	
None to some of the time	61 (32.6%)
Most or all of the time	128 (67.4%)
**Someone to turn to for suggestions about how to deal with a personal problem**	
None to some of the time	58 (31.1%)
Most or all of the time	131 (68.9%)
**Someone who understands your problems**	
None to some of the time	70 (37.4%)
Most or all of the time	119 (62.6%)
**Someone to help you if you were confined to bed**	
None to some of the time	72 (37.9%)
Most or all of the time	117 (62.1%)
**Someone to take you to the doctor if you need it**	
None to some of the time	71 (37.4%)
Most or all of the time	118(62.6%)
**Someone to prepare your meals if you were unable to do it yourself**	
None to some of the time	72 (37.9%)
Most or all of the time	117 (62.1%)
**Someone to help with daily chores if you were sick**	
None to some of the time	79 (41.6%)
Most or all of the time	110 (58.4%)
**Someone who shows you love and affection**	
None to some of the time	42 (22.1%)
Most or all of the time	147 (77.9%)
**Someone to love and make you feel wanted**	
None to some of the time	37 (19.5%)
Most or all of the time	152 (80.5%)
**Someone who hugs you**	
None to some of the time	46 (24.2%)
Most or all of the time	143 (75.8%)
**Someone to have a good time with**	
None to some of the time	73 (38.4%)
Most or all of the time	116 (61.6%)
**Someone to get together with for relaxation**	
None to some of the time	69 (36.3%)
Most or all of the time	120 (63.7%)
**Someone to do something enjoyable with**	
None to some of the time	79 (41.6%)
Most or all of the time	110 (58.4%)
**Someone to do things with to help you get your mind off things**	
None to some of the time	71 (37.4%)
Most or all of the time	118 (62.6%)

**Table 4:** Clinical characteristics and outcomes of patients who did not complete the PHQ-9 survey 3 months post-trauma (N = 56).

Variable	Value
**Injury Severity Score (Mean ± SD)**	15.9 ± 7.8
**GCS at the scene (Mean ± SD)**	14.1 ± 2.2
**GCS in trauma unit (Mean ± SD)**	13.8 ± 3.2
**GCS after 1 h in trauma (Mean ± SD)**	13.5 ± 3.8
**Spine AIS** (Mean ± SD)	2.4 ± 0.8
Cervical spine injury	17 (30.4%)
Thoracic spine injury	26 (46.4%)
Lumbar spine injury	0(0%)
Sacral	2 (3.6%)
**Spinal surgery**	13 (23.2%)
**Neurological deficit**	6 (10.7%)
**Psychiatry referral**	7 (12.5%)
**Rehabilitation Referral**	17 (30.4%)
**Psychology referral**	13 (23.2%)
**LOS**	8 (IQR 5–16)

SD: Standard deviation; AIS: Abbreviated Injury Scale; LOS: Length of Stay; IQR: Interquartile Range.

**Table 5:** Patient Health Questionnaire-9 (PHQ-9) item-level responses at two weeks and three months following traumatic spinal injury (N = 133).

PHQ-9 Question & Response Category	Post 2 Weeks Trauma	Post 3 Months Trauma	*p*-Value
**1.** **Little interest or pleasure in doing things**			0.140
Not at all	102 (76.7%)	118 (88.7%)	
Several days	15 (11.3%)	6 (4.5%)	
More than half the days	9 (6.8%)	5 (3.8%)	
Nearly every day	7 (5.3%)	4 (3.0%)	
**2.** **Feeling down, depressed, or hopeless**			0.016
Not at all	72 (54.1%)	98 (73.7%)	
Several days	29 (21.8%)	19 (14.3%)	
More than half the days	21 (15.8%)	11 (8.3%)	
Nearly every day	11 (8.3%)	5 (3.8%)	
**3.** **Trouble falling or staying asleep or sleeping too much**			0.001
Not at all	68 (51.1%)	102 (76.7%)	
Several days	29 (21.8%)	14 (10.5%)	
More than half the days	15 (11.3%)	11 (8.3%)	
Nearly every day	21 (15.8%)	6 (4.5%)	
**4.** **Feeling tired or having little energy**			0.052
Not at all	75 (56.4%)	95 (71.4%)	
Several days	31 (23.3%)	24 (18.0%)	
More than half the days	11 (8.3%)	8 (6.0%)	
Nearly every day	16 (12.0%)	6 (4.5%)	
**5.** **Poor appetite or overeating**			0.261
Not at all	98 (77.4%)	110 (82.7%)	
Several days	11 (8.3%)	14 (10.5%)	
More than half the days	14 (10.5%)	8 (6.0%)	
Nearly every day	10 (7.5%)	1 (0.8%)	
**6.** **Feeling bad about yourself or that you are a failure or have let yourself or your family down**			0.610
Not at all	103 (77.4%)	109 (82.0%)	
Several days	20 (15.0%)	14 (10.5%)	
More than half the days	6 (4.5%)	4 (3.0%)	
Nearly every day	4 (3.0%)	6 (4.5%)	
**7.** **Trouble concentrating on things, such as reading the newspaper or watching television**			0.419
Not at all	106 (79.7%)	115 (86.5%)	
Several days	11 (8.3%)	9 (6.8%)	
More than half the days	11 (8.3%)	4 (3.0%)	
Nearly every day	5 (3.8%)	5 (3.8%)	
**8.** **Moving or speaking so slowly that other people could have noticed. Or the opposite—being so fidgety or restless that you have been moving around a lot more than usual.**			0.127
Not at all	105 (78.9%)	122 (91.7%)	
Several days	11 (8.3%)	4 (3.0%)	
More than half the days	9 (6.8%)	1 (0.8%)	
Nearly every day	8 (6.0%)	6 (4.5%)	
**9.** **Thoughts that you would be better off dead or hurting yourself**			0.327
Not at all	121 (91.0%)	127 (95.5%)	
Several days	9 (6.8%)	4 (3.0%)	
More than half the days	1 (0.8%)	2 (1.5%)	
Nearly every day	2 (1.5%)	0	
**McNemar test for paired categorical data**.			

**Table 6:** Baseline PHQ-9 item responses at 2 weeks comparing patients who completed follow-up and those lost to follow-up.

PHQ-9 Question & Response Category	Completed (N = 133)	Lost (n = 56)	*p*-Value
**1.** **Little interest or pleasure in doing things**			0.24
Not at all	102 (76.7%)	42 (75.0%)	
Several days	15 (11.3%)	10 (17.9%)	
More than half the days	9 (6.8%)	4 (7.1%)	
Nearly every day	7 (5.3%)	0 (0%)	
**2.** **Feeling down, depressed, or hopeless**			0.25
Not at all	72 (54.1%)	36 (64.3%)	
Several days	29 (21.8%)	9 (16.1%)	
More than half the days	21 (15.8%)	10 (17.9%)	
Nearly every day	11 (8.3%)	1 (1.7%)	
**3.** **Trouble falling or staying asleep or sleeping too much**			0.53
Not at all	68 (51.1%)	35 (62.5%)	
Several days	29 (21.8%)	10 (17.9%)	
More than half the days	15 (11.3%)	4 (7.1%)	
Nearly every day	21 (15.8%)	7 (12.5%)	
**4.** **Feeling tired or having little energy**			0.20
Not at all	75 (56.4%)	38 (67.9%)	
Several days	31 (23.3%)	9 (16.1%)	
More than half the days	11 (8.3%)	1 (1.7%)	
Nearly every day	16 (12.0%)	8 (14.3%)	
**5.** **Poor appetite or overeating**			0.44
Not at all	98 (77.4%)	46 (82.2%)	
Several days	11 (8.3%)	4 (7.1%)	
More than half the days	14 (10.5%)	2 (3.6%)	
Nearly every day	10 (7.5%)	4 (7.1%)	
**6.** **Feeling bad about yourself or that you are a failure or have let yourself or your family down**			0.07
Not at all	103 (77.4%)	37 (66.0%)	
Several days	20 (15.0%)	10 (17.9%)	
More than half the days	6 (4.5%)	2 (3.6%)	
Nearly every day	4 (3.0%)	7 (12.5%)	
**7.** **Trouble concentrating on things, such as reading the newspaper or watching television**			0.59
Not at all	106 (79.7%)	48 (85.8%)	
Several days	11 (8.3%)	5 (8.9%)	
More than half the days	11 (8.3%)	2 (3.6%)	
Nearly every day	5 (3.8%)	1 (1.7%)	
**8.** **Moving or speaking so slowly that other people could have noticed. Or the opposite—being so fidgety or restless that you have been moving around a lot more than usual.**			0.20
Not at all	105 (78.9%)	49 (87.5%)	
Several days	11 (8.3%)	3 (5.4%)	
More than half the days	9 (6.8%)	0 (0%)	
Nearly every day	8 (6.0%)	4 (7.1%)	
**9.** **Thoughts that you would be better off dead or hurting yourself**			0.68
Not at all	121 (91.0%)	50 (89.4%)	
Several days	9 (6.8%)	5 (8.9%)	
More than half the days	1 (0.8%)	1 (1.7%)	
Nearly every day	2 (1.5%)	0 (0%)	
**PHQ-9:** Patient Health Questionnaire-9; chi-square test			

**Table 7:** Paired statistics and effect size.

Paired Statistic	Value
Mean PHQ-9 (2 weeks)	4.6
Mean PHQ-9 (3 months)	2.5
Mean difference	2.098
Standard deviation (SD) of difference	4.985
Standard error	0.432
95% Confidence interval	1.243–2.953
t value	4.853
df (degree of freedom)	132
*p*-value	<0.001

Calculate Cohen’s d: Cohen’s d = Mean difference/SD difference: d = 2.098/4.985 = 0.421.

## Data Availability

The data used in this study are available upon request from the corresponding author. However, data sharing is subject to institutional review board approval and applicable data privacy regulations.

## Abbreviations

SCI	spinal cord injury
PHQ	patient health questionnaire
ISS	injury severity score
